# Modeling connectivity to identify current and future anthropogenic barriers to movement of large carnivores: A case study in the American Southwest

**DOI:** 10.1002/ece3.2939

**Published:** 2017-04-18

**Authors:** Meredith L. McClure, Brett G. Dickson, Kerry L. Nicholson

**Affiliations:** ^1^ Conservation Science PartnersTruckeeCAUSA; ^2^ University of ArizonaTucsonArizona

**Keywords:** habitat fragmentation, highway mitigation, land use change, land use planning, movement ecology, permeability, road ecology, space use, urbanization, wildlife conflict

## Abstract

This study sought to identify critical areas for puma (*Puma concolor*) movement across the state of Arizona in the American Southwest and to identify those most likely to be impacted by current and future human land uses, particularly expanding urban development and associated increases in traffic volume. Human populations in this region are expanding rapidly, with the potential for urban centers and busy roads to increasingly act as barriers to demographic and genetic connectivity of large‐bodied, wide‐ranging carnivores such as pumas, whose long‐distance movements are likely to bring them into contact with human land uses and whose low tolerance both for and from humans may put them at risk unless opportunities for safe passage through or around human‐modified landscapes are present. Brownian bridge movement models based on global positioning system collar data collected during bouts of active movement and linear mixed models were used to model habitat quality for puma movement; then, a wall‐to‐wall application of circuit theory models was used to produce a continuous statewide estimate of connectivity for puma movement and to identify pinch points, or bottlenecks, that may be most at risk of impacts from current and future traffic volume and expanding development. Rugged, shrub‐ and scrub‐dominated regions were highlighted as those offering high quality movement habitat for pumas, and pinch points with the greatest potential impacts from expanding development and traffic, although widely distributed, were particularly prominent to the north and east of the city of Phoenix and along interstate highways in the western portion of the state. These pinch points likely constitute important conservation opportunities, where barriers to movement may cause disproportionate loss of connectivity, but also where actions such as placement of wildlife crossing structures or conservation easements could enhance connectivity and prevent detrimental impacts before they occur.

## 1 Introduction

As natural habitats become increasingly fragmented by human land use and activity, maintaining permeable landscapes that support the dispersal processes that enable demographic and genetic connectivity among wildlife populations becomes increasingly important (McRae, Beier, Dewald, Huynh, & Keim, 2005; Sawyer et al., 2013). This is particularly true for species that typically require large home ranges to meet resource needs and whose dispersal movements occur over broad spatial extents. Wide‐ranging species, such as large carnivores, are more likely to experience negative population‐level effects of habitat fragmentation and to exhibit low tolerance for human activity (Crooks, Burdett, Theobald, Rondinini, & Boitani, 2011). Concomitantly, these same species are often subject to persecution from humans (Kellert, Black, Rush, & Bath, 1996). Thus, access to safe passages through or around human‐modified landscapes is critical to maintaining connectivity among populations that persist in human‐dominated landscapes.

Increasing urbanization and the expansion of exurban (i.e., formerly rural) areas can threaten the viability of animal populations occupying adjacent habitats (Hansen et al., 2005; Riley et al., 2003). Beyond the fragmentation of natural landscapes, exurban development can lead to loss of key habitat features (e.g., forage and cover; Parmenter et al., 2003), reduced fitness among individuals (Hansen et al., 2005), and increased conflicts with humans (Kretser, Sullivan, & Knuth, 2008). Large carnivores may be particularly sensitive to these impacts (Crooks, 2002; Goad, Pejchar, Reed, & Knight, 2014). Urbanization and exurban growth also brings increased vehicle traffic volumes, which can be a source of direct mortality (Gunther et al., 2004; Mumme, Schoech, Woolfenden, & Fitzpatrick, 2000). High traffic volume may also induce road avoidance behavior (Northrup et al., 2012) and may cause roads to become complete barriers to movement of some species, with detrimental impacts on population demographics (Gibbs & Steen, 2005; Mumme et al., 2000) or long‐term population persistence (Epps, Palsboll, & Wehausen, 2005; Sweanor, Logan, & Hornocker, 2000).

Pumas (*Puma concolor*) are well distributed throughout the Desert Southwest of the United States, including Arizona (NatureServe 2015). In the northern portion of the state, pumas are considered common across the variety of vegetation communities that also are occupied by their principal prey, namely mule deer (*Odocoileus hemionus*) (Logan & Sweanor, 2001). In the southern portion of the state, pumas tend to occupy more mountainous or rugged areas that otherwise are surrounded by desert basins (McRae et al., 2005). Although pumas may infrequently traverse these basin features (Nicholson, 2009; Sweanor et al., 2000), these areas tend to also be readily used and impacted by humans and may increase risks associated with movement. Pumas are known to be sensitive to the presence of human structures (Beier, 1995; Wilmers et al., 2013), roads (Beier & Barrett, 1993; Dickson, Jenness, & Beier, 2005; Sweanor et al., 2000), traffic (Alexander & Waters, 2000; Alexander, Waters, & Paquet, 2005; Schwab & Zandbergen, 2011), and other human activity (Beier, 1995; Morrison, Boyce, Nielsen, & Bacon, 2014). As human communities in the Southwest continue to expand more quickly than other regions of the United States (U.S. Census Bureau 2015), increased urban growth and exurban development, increased traffic volumes, expanding utility infrastructure to meet increased energy demands, and heightened border security and interdiction activities (Preston, 2013) will likely bring pumas into more frequent contact with human‐modified landscapes and barriers to movement. Identification of critical pathways that may be most at risk from human land use and activity would support proactive mitigation of these impacts.

The principal objectives of this study were to (1) estimate and map habitat quality and connectivity for puma movement throughout the state of Arizona and (2) identify places (e.g., “pinch points”) with high potential connectivity that may be at risk of becoming severed due to increasing development pressure and associated increases in traffic volume. Here, habitat quality refers not to the general condition of the landscape, but to the relative frequency or probability of use for puma movement (after Dickson, Sesnie, Fleishman, & Dobkin, 2013). This analysis leverages knowledge from previous studies of puma habitat suitability (Burdett et al., 2010; Dickson et al., 2005; Wilmers et al., 2013) and connectivity (Dickson, Roemer, McRae, & Rundall, 2013) in portions of the western United States, but is the first to use empirically based, high spatial resolution movement data to map continuous habitat quality and connectivity for pumas over a large region. It also leverages existing modeling techniques (i.e., Brownian bridge movement models [BBMMs], circuit theory models), but integrates these techniques in a unique way that offers novel benefits. Specifically, this approach develops estimates of habitat quality that are explicitly tied to the processes of movement and dispersal requiring no incongruous assumptions about the relationship between habitat quality and resistance to movement (a common problem in connectivity modeling studies; Zeller, McGarigal, & Whiteley, 2012), and does not rely on subjective definitions of the location or configuration of discrete habitat patches (e.g., Dickson, Roemer, et al., 2013; Dickson, Sesnie, et al., 2013). The resulting connectivity model is used to assess current and potential threats to puma movement resulting from human land use and activity. The goal of this study was to provide practical information that could help to guide planning efforts concerned with the conservation of pumas and their habitat in a rapidly developing region of the West, while also advancing current connectivity modeling methodology in ways that could be extended to other species and landscapes.

## 2 Materials and methods

### 2.1 Study area

The study area encompassed the state of Arizona in the Desert Southwest of the United States (area = 295,289 km^2^). A wide variety of vegetation communities was present, including ponderosa pine (*Pinus ponderosa*)‐dominated forest types across the north and desert scrub and shrub and woodland across much of the rest of state. The topography of the state was rugged, and elevations ranged from 25 m in the southwest to 3,851 m at the top of Humphreys Peak in the north (mean elevation = 1,284, *SD* = 642). Numerous, isolated mountain ranges (“Sky Islands”) were present throughout the southern portion of the state, which were dominated by mixed‐coniferous and deciduous forests at elevations >2,000 m. This diversity of natural cover and terrain types supported the movement patterns of pumas and their primary prey, mule deer. Most (>82%) of Arizona's land was publicly owned (USGS National Gap Analysis Program 2007). As of July 2014, the human population of Arizona was >6.7 million, and Phoenix (1.54 million), Tucson (527,972), Flagstaff (68,785), Prescott (40,958), and Payson (15,245) were among the largest urban or suburban centers (U.S. Census Bureau 2015). Major interstate highways included I‐8, I‐10, I‐17, I‐19, and I‐40, totaling >1,800 km in length (Arizona Department of Transportation 2015). Arizona shared a border with Mexico that extended >3,100 km, and a presumably large portion of this border acted as a barrier to wildlife movement due to construction of the Mexico‐U.S. border fence and other border security measures (Preston, 2013).

### 2.2 Location data

Analyses were based on existing global positioning system (GPS) collar location data from 28 pumas captured and monitored by the Arizona Game and Fish Department (AGFD) between August 2005 and March 2008 in the areas surrounding Payson, Prescott, and Tucson, Arizona (Nicholson, 2009). Capture efforts were focused on mountain ranges adjacent (<10 km) to urban areas because the objective of the original study was to examine the ecology and spatial movements of mountain lions near urban areas. Thirty individuals of at least 2 years of age were captured and fitted with Spread Spectrum GPS collars (Telonics, Mesa, Arizona) programmed to obtain satellite locations every 4.15 hr for pumas near Tucson or every 7 hr for pumas near Payson and Prescott. One collar was not retrieved, and data from another collar that collected only 73 locations were excluded. Locations with positional dilution of precision (PDOP) >10 were also excluded, yielding a total of 30,209 locations from 28 individuals. In order to estimate movement probability based only on bouts of active movement, we identified locations likely to be associated with den or kill sites as those within 200 m of adjacent locations and excluded them (Anderson & Lindzey, 2003; Knopff, Knopff, Warren, & Boyce, 2009), leaving 20,303 locations for use in analyses (Table S1).

### 2.3 Modeling probability of movement

**Figure 1:**
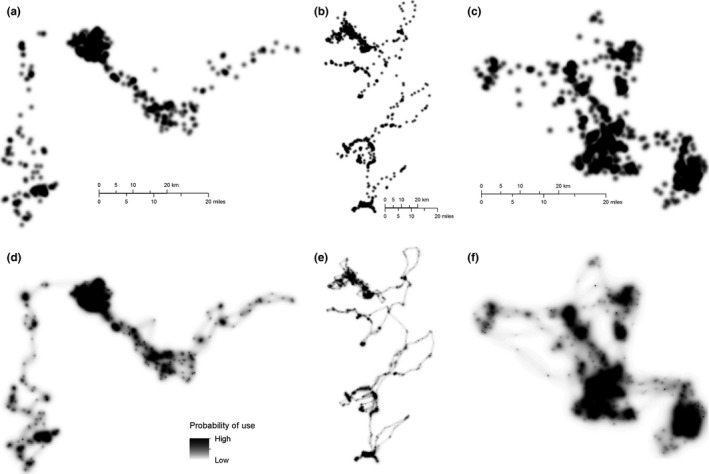
Estimated space use of three representative individual pumas. (a–c) Kernel density estimates using the adehabitat package for R (Calenge, 2006) (bivariate normal kernel, default smoothing method with smoothing parameter = 500). (d–f) Brownian bridge movement models calculated using the Brownian Bridge movement models (BBMM) package for R (Brownian motion variance parameter = 102.75 (d), 243.71 (e), 102.35 (f); location error = 26.2 m; maximum lag = 24 hr). Kernel density function parameters were selected to produce surfaces as similar as possible to the BBMM surfaces for comparison

Brownian Bridge movement models were used to estimate the probability of each individual moving through a given area between locations (Horne, Garton, Krone, & Lewis, 2007). BBMMs are conditioned on distance, elapsed time, and a parameter estimating an individual's mobility between locations, allowing them to estimate space use along movement paths (Figure 1d–f). In contrast, traditional utilization distributions, which use kernel density functions to estimate space use (e.g., Millspaugh & Nielson, 2006), do not incorporate information about the order of, or elapsed time between locations and therefore simply estimate point density (Figure 1a–c). BBMMs were estimated for each individual using the “brownian.bridge” function in the BBMM package (Nielson, Sawyer, & McDonald, 2013) for R (R Core Team 2013). BBMMs assumed an average location error of 26.2 m (95% circular error probable) for GPS locations with PDOP < 10 (D'Eon & Delparte, 2005). While this value is not specific to the collars used in this study, it provides a reasonable location error estimate and produces more conservative BBMM outputs than would be obtained by simply excluding an error term. The maximum allowed time between consecutive locations was 24 hr. If the time between consecutive locations exceeded this limit (~1% of observed locations), then a Brownian bridge was not estimated between the location pair because large gaps can artificially inflate or deflate the Brownian motion variance and may bias estimates of movement probability. The spatial extent of each BBMM was defined to include all areas with >.00001 probability of movement. This step was intended to reduce computing time and output file size (Nielson et al., 2013), but also ensured that the bounds of the BBMM surface were defined in relation to the observed movement path rather than the rectangular map extent.

### 2.4 Selecting habitat variables

Based on recent literature on puma habitat selection and space use in the West, habitat variables that were hypothesized to impact patterns of space use during bouts of active movement and connectivity in Arizona were identified, representing topography, availability of water, vegetation, and human modification of the landscape (Table S2). All variables were derived as 30‐m resolution spatial data layers across the state and then aggregated to a final resolution of 270 m to match the coarser resolution of the preexisting human modification data layer. All values were standardized and rescaled prior to model fitting and averaging (Cade, 2015). Screening for pairwise correlations was based on Pearson's correlation coefficients and for multicollinearity based on variance inflation factors (VIFs). All Pearson's coefficients were ≤0.5 and all VIFs were <2.5, well below arbitrary but common cutoff values of 0.7 and 4.0, respectively, and were therefore retained for further analysis.

We derived ruggedness and a topographic position index (TPI) from 30‐m resolution USGS digital elevation models (USGS 2012). Ruggedness was calculated as the standard deviation of slope values in a 9 × 9‐cell moving window (270 m × 270‐m neighborhood) around each 30‐m map cell. Topographic position was calculated as the elevation of each 30‐m focal cell minus the mean elevation of cells within a given distance. Because calculations of TPI are sensitive to scale and pumas may respond to topography at a variety of spatial scales, a multiscale TPI was used, which was calculated as a composite across five neighborhood sizes (720–21,870‐m; Theobald, Harrison‐Atlas, Monahan, & Albano, 2015).

Using the National Hydrography Dataset (USGS 2014), water features expected to provide reliable sources of water for pumas were selected, including springs and seeps; perennial streams and rivers; perennial lakes and ponds; swamps and marshes; and reservoirs designated as serving water storage and aquaculture purposes. Spatial data on “wildlife waters” maintained as supplemental water sources by AGFD were added to these features. Distance to the nearest water feature was then calculated at a 30‐m resolution.

Variables representing availability of forest and woodland cover, riparian cover, and shrub and scrub cover were derived from the Southwest Regional Gap Analysis Project dataset (USGS National Gap Analysis Program 2004). First, vegetation classes comprising each of these cover types were selected. Using a 9 × 9‐cell moving window, the proportion of the moving window's area dominated by each cover type around each 30‐m map cell was then calculated.

A human modification index derived nationally at 270‐m resolution by Theobald (2010) was used to represent the degree of human impact on the landscape. This index integrates national datasets on land cover, housing density, roads, and highway traffic volume to estimate the proportion of natural land cover—or conversely, the proportion of landscapes that are human‐modified—within spatial neighborhoods of multiple sizes. The final multiscale human modification index represents the arithmetic mean across scales.

### 2.5 Modeling habitat quality for movement

The relationship between each BBMM and the habitat variables described above was modeled to estimate habitat quality for movement. Each BBMM surface was first buffered by a distance equal to the greatest step length between consecutive locations observed for each individual. Buffered areas were intended to capture landscape features that were available for pumas to move through but that may have been avoided. The same number of random points was then sampled from buffered individual BBMMs as the number of locations used to estimate them (e.g., Willems & Hill, 2009). Habitat quality for movement was then estimated using linear mixed models (LMMs) and multimodel inference (Burnham & Anderson, 2002). LMMs were fitted using individual as a subject‐level random effect and used an exponential spatial covariance structure to account for residual spatial autocorrelation (Dormann et al., 2007). All subsets of a global model that contained linear terms (the fixed effects) for the habitat variables described above, as well as a quadratic term for ruggedness, were fitted. The global model also included fixed‐effect indicators representing sex (male vs. female), age class (young adult (2–3 years) vs. adult (≥4 years)), and area of capture (Payson vs. Prescott vs. Tucson).

Maximum likelihood and values of Akaike's Information Criterion (AIC; Burnham & Anderson, 2002) were used to determine how well the global model approximated the data, compared to a null model that included only a subject‐level random effect. A global model with an AIC value >10.0 units lower than the null model was considered to provide a good approximation of the data (Burnham & Anderson, 2002). For each environmental variable (i.e., fixed effect), model‐averaged regression coefficients (*β* ${}^{≃}$), unconditional standard errors, and (*w*_*+*_) as a measure of the weight of evidence in favor of a given variable (Burnham & Anderson, 2002; Lukacs, Burnham, & Anderson, 2006) were derived. The empirical Huber‐White “sandwich” estimator was used to compute the variance‐covariance matrix of fixed‐effects parameters (Wooldridge, 2009). All analyses were conducted in SAS (v9.3; SAS Institute, Inc., Cary, North Carolina, USA) and the R statistical environment (v3.0.3; R Core Team 2013).

Habitat quality for puma movement was mapped continuously across the state of Arizona using the model‐averaged regression coefficients. This map was used to represent landscape conductance (i.e., the reciprocal of landscape resistance, such that higher conductance values denote greater ease of movement) for use in modeling connectivity, after assigning the minimum habitat quality value to the Colorado River, the Central Arizona Project canal, and open water bodies, including lakes and reservoirs. The assumption of a simple inverse relationship between habitat quality and resistance has been questioned, and exploration of alternative transformations of quality to resistance has been encouraged (e.g., Mateo‐Sanchez et al., 2015; Zeller et al., 2012). However, because movement habitat quality was estimated directly from puma space use during bouts of active movement in this study, it is logically consistent in this case to directly equate habitat quality with conductance and, consequently, the inverse of resistance.

### 2.6 Modeling statewide connectivity

Circuitscape software (v4.0.3; McRae, Shah, & Mohapatra, 2013) was used to estimate omnidirectional connectivity across Arizona. Circuit‐based models apply concepts related to flow of charge through an electrical circuit to the movement of individuals through a landscape (McRae, Dickson, Keitt, & Shah, 2008). Cells in a landscape are treated as electrical nodes connected to neighboring cells by resistors, with resistance values defined by a model of the landscape's resistance to movement (or, inversely, conductance). When an electrical charge passes through the circuit from a source location to a destination location, current values at each cell in the landscape represent the probability of a random walker passing through the cell as it moves from the source to the destination. Higher current densities are found at pinch points, where many potential paths condense to pass through a narrow area because few alternative paths are available (McRae et al., 2008).

Most previous applications of circuit theory to modeling habitat connectivity have been implemented using “cores” of high quality habitat patches to estimate current flow between source and destination pairs (e.g., Poor, Loucks, Jakes, & Urban, 2012; Dickson, Roemer, et al., 2013). However, inferences regarding spatial patterns and degree of connectivity across a given area of interest may be sensitive to an arbitrary designation of what constitutes a core, as well as the location and extent of cores. Here, an alternative “omnidirectional” approach (Anderson, Clark, & Olivero, 2012; Pelletier et al., 2014) was applied to produce a continuous, “wall‐to‐wall” estimate of connectivity across the landscape in any direction.

Two pairwise model runs were implemented in Circuitscape, first designating single pixel‐wide (270 m) strips along the east and west boundaries of the rectangular map extent encompassing the state of Arizona as parallel source and destination regions, then designating the north and south rectangular extent boundaries as the source‐destination pair. Cells in the areas between the rectangular map extent and the irregular state boundary were randomly filled with values from a normal distribution approximating the observed distribution of modeled conductance values (after Koen, Garroway, Wilson, & Bowman, 2010). This allowed current to “percolate” evenly into and out of the state boundaries from the source region to the destination region without introducing edge effects. The east‐west and north‐south model runs were then summed to estimate omnidirectional connectivity across the state. Pelletier et al. (2014) demonstrated that inclusion of additional runs in arbitrary oblique directions did not discernably affect the spatial connectivity patterns seen.

### 2.7 Assessing potential land use impacts on puma connectivity pinch points

Lastly, locations where current and future human land use and activity may be most likely to adversely impact high levels of connectivity and present potential barriers to puma movements were identified. These “at‐risk” pinch points may highlight important conservation opportunities, where imposing a barrier to movement would cause disproportionate loss of connectivity. This study made use of existing datasets to assess risk to pinch points, defined as locations in the 95th percentile of current flow (connectivity values), in the context of (1) projected change in the prevalence of impervious surface (i.e., surfaces covered by impenetrable materials such as asphalt and concrete) and (2) current and projected change in vehicle traffic on roads.

Data representing the extent of impervious surface in 2010 and projections for 2030, generated by the Integrated Climate and Land Use Scenarios (ICLUS) model (U.S. Environmental Protection Agency 2010), were obtained. Although other forms of land cover modification may also restrict puma movements, we focused on conversions of vegetative land cover to artificial structures (i.e., pavement, building) as those most likely to generate absolute barriers to puma movement. ICLUS datasets offer continuous statewide coverage of percent impervious surface cover at 1‐km resolution. Projections that assume a baseline scenario were used, in which future trajectories are consistent with current human demographic rates. Because the models of habitat quality and connectivity produced here already captured puma response to current levels of human modification, which include the presence of impervious surface, only change in percent impervious surface cover between 2010 and 2030 was assessed to identify pinch points that are expected to be impacted most by future development. Within map cells with expected increases in impervious surface, pinch points that may experience the highest impacts were selected as those in the 90th percentile of projected impervious surface increase.

Statewide annual average daily traffic (AADT) data for 2013 were also obtained, as well as projections for 2030 from the Arizona Department of Transportation. 2013 traffic volume was overlaid on our statewide connectivity layer to identify mile‐marker locations where pinch points are intersected by roads with high traffic volumes that may be expected to deter or present a barrier to puma movement (>5,000 vehicles/day) (Alexander et al., 2005). Because there is typically a drop in otherwise high current values at the precise location where a pinch point intersects a high‐resistance road, mile markers were assigned the highest current value within a 1‐km radius.

Projected change in traffic volume was calculated as the difference between 2010 observed volume and 2030 projected volume. Pinch points were then identified where puma movements were not expected to be deterred by current high traffic volumes (<3,000 vehicles/day) or were expected to be marginally impacted (3,000–5,000 vehicles/day), but where these threshold traffic volumes are expected to be exceeded in the future (Alexander & Waters, 2000; Alexander et al., 2005).

## 3 Results

### 3.1 Patterns in puma movement probability

BBMMs clearly highlighted probabilistic travel routes between consecutive locations during directional movements that would have been missed using traditional UD methods, which do not incorporate information about the sequence of and time between locations (e.g., Figure 1). Individual movement patterns varied in terms of area covered and relative density. Strong negative coefficients in the model‐averaged movement habitat quality result for indicators representing young adults and males suggest that male space use (or probability of movement through any given location) was less concentrated (more diffuse) than that of females and that movements of young adults were less concentrated than those of adults (Table 1). Space use by pumas collared near Tucson and, particularly, around Prescott, was estimated to be less concentrated than pumas collared near Payson.

**Table 1:** Global model of puma movement habitat quality. Weights of evidence (*w*_*+*_), model‐averaged regression coefficients (*β* ${}^{≃}$), and unconditional standard errors (*SE*) for variables used to estimate probability of puma movement are included

Variable	*w*_*+*_	(*β* ${}^{≃}$)	*SE*
Young adult indicator	1.000	−1.512	.423
Male indicator	1.000	−2.833	.911
Prescott indicator	1.000	−1.554	.732
Tucson indicator	1.000	−.793	.756
Ruggedness	.999	.583	.156
Percent shrub/scrub cover	.945	.311	.208
Human modification	.728	−.190	.129
Percent riparian cover	.396	.028	.044
Ruggedness^2^	.375	−.031	.064
Distance to water	.339	−.080	.154
Percent forest cover	.287	−.018	.072
Topographic position	.272	−.003	.070
Intercept	NA	6.833	1.033

^2^Quadratic term for ruggedness variable.

### 3.2 Habitat quality for movement

The global model had an AIC value 35 units lower (i.e., better) than the null model, suggesting that it approximated the data well. Weights of evidence indicated that increasing terrain ruggedness and increasing shrub/scrub cover were the strongest predictors of puma habitat quality for movement (*w*_*+*_ = .999 and .945, respectively), followed by decreasing degree of human modification (*w*_*+*_ = .728) (Table 1). However, there is some evidence that movement habitat quality may decline in the most rugged terrain (*w*_*+*_ = .375). There is also some evidence that movement habitat quality may increase with greater riparian cover (*w*_*+*_ = .396) and closer to reliable sources of water (*w*_*+*_ = .339). Forest/woodland cover and topographic position were less important in predicting habitat quality for movement (*w*_*+*_ = .287 and .272, respectively).

### 3.3 Statewide patterns of puma habitat connectivity

**Figure 2:**
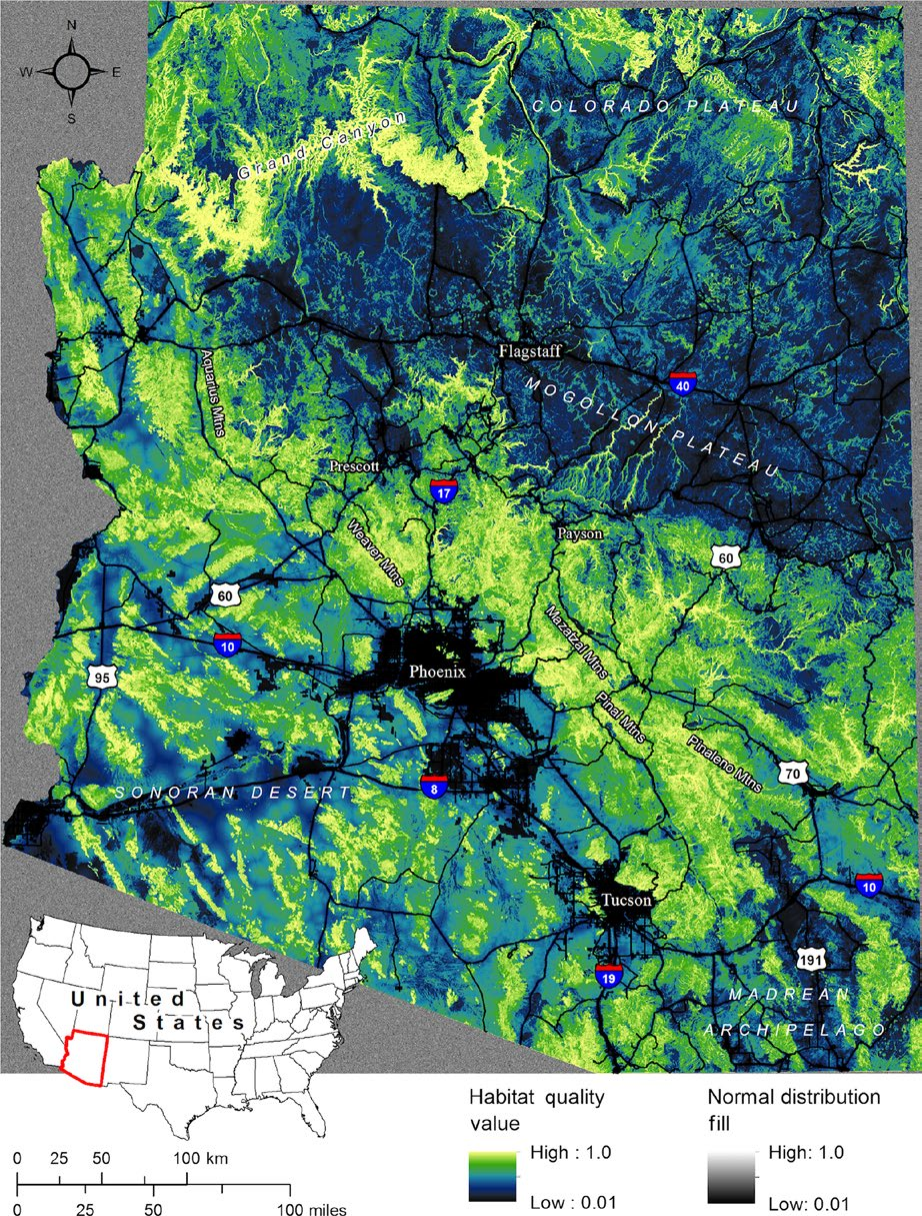
Map of predicted habitat quality for puma movement across Arizona. Map edges were filled with values randomly selected from a normal distribution of habitat quality values to avoid edge effects when running wall‐to‐wall circuit theory models across the irregularly shaped state of Arizona, located in the southwest United States (inset). Quality values are displayed using a histogram‐equalized classification

**Figure 3:**
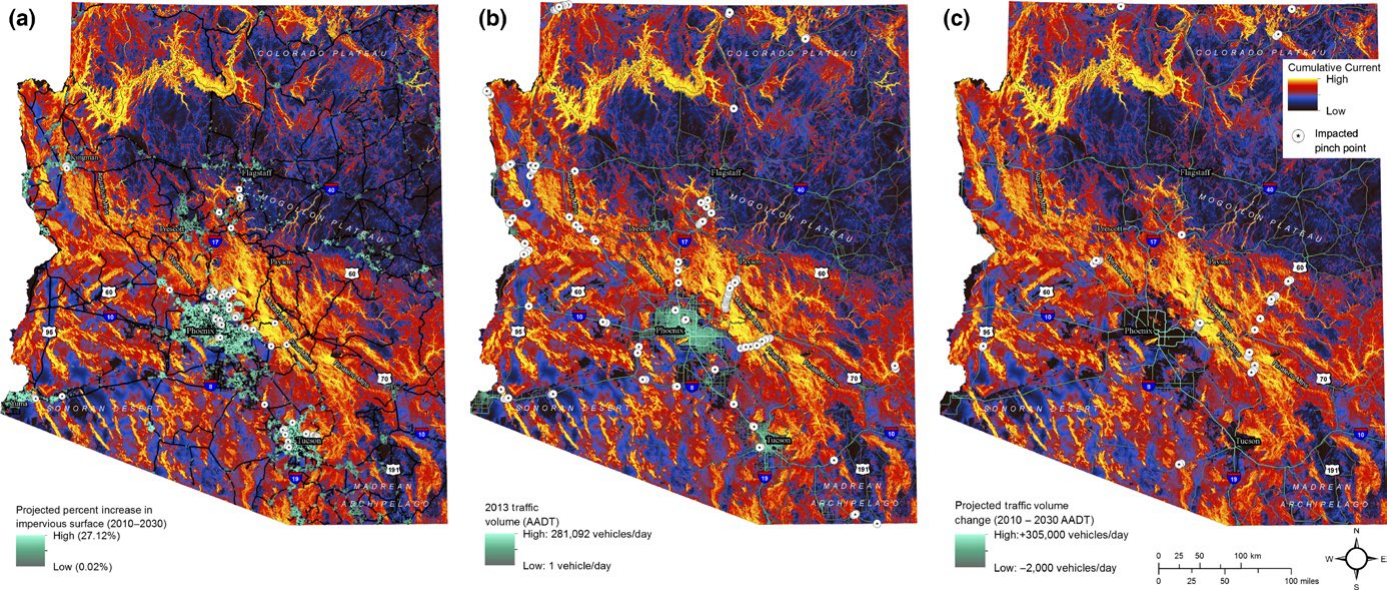
Maps of predicted cumulative current flow (connectivity value) for puma movement across Arizona, overlaid with (a) projected change in percent impervious surface between 2010 and 2030, (b) 2013 annual average daily traffic volume (AADT), and (c) projected change in AADT volume between 2010 and 2030 is overlaid. Current values are displayed using a histogram‐equalized classification; impervious surface and AADT values are displayed using a geometric classification. Full‐resolution image is available on Dryad; see Data Accessibility

As expected, omnidirectional patterns of connectivity across the state closely mirror patterns of movement habitat quality (Figure 2), with broad areas of high quality and high movement probability associated with more rugged, shrub‐ and scrub‐dominated regions (e.g., the greater Grand Canyon area and the Aquarius, Weaver, Mazatzal, Pinal, and Pinaleno Mountains) stretching east to west across the state (Figures 2 and 3). The model also highlighted more fragmented patches of high quality habitat where connectivity was high in the Madrean Archipelago, also known as the Sky Islands, in the southeastern portion of the state. Low connectivity was associated with flat, open areas (e.g., the Mogollon Plateau), and high levels of human modification (i.e., urban areas and major highways). Differences between the habitat quality and connectivity models were apparent where connectivity was influenced by water features that were assigned low conductance. At finer scales, the model highlights pinch points where routes within broad areas of high quality habitat may be of particular importance, as well as constricted routes where movement is predicted to be funneled through areas of low habitat quality or across barriers (Figures 3 and S1–S3).

### 3.4 Current and future impacts of development and traffic on pinch points

We identified pinch points most likely to be impacted by increases in impervious surface (Figure 3a), current traffic volume (Figure 3b), and increases in traffic volume (Figure 3c). The presence of impervious surface is expected to increase considerably in and around Phoenix and Tucson (up to 27%/km^2^), as well as in and adjacent to other urban centers, particularly as development increases along major highways extending out of cities (Figure 3a). Pinch points that may be most impacted by increasing impervious surface were found clustered along the northeast edge of Phoenix, at the western and northeastern edges of Tucson, south of Flagstaff, and adjacent to smaller population centers (Figures 3a and S1; Table S3).

The highest current traffic volumes are found within urban areas (e.g., Phoenix, Tucson, Yuma), but are also heavy along many interstate, U.S., and state highways that connect these urban centers and cross potential connectivity pinch points throughout the state (Figures 3b and S2; Table S4). Traffic volume is expected to increase most along highways within the greater Phoenix area (up to 305,000 additional vehicles/day), but substantial increases are also anticipated along I‐10 between Phoenix and Tucson (15,500–96,000 additional vehicles/day), along I‐17 between Phoenix and Flagstaff (8,500–50,000 additional vehicles/day), and along other major highways (Figure 3c). Sites at which traffic volumes are currently expected to have little impact (<3,000 vehicles/day) or marginal impact (3,000–5,000 vehicles/day), but that are projected to exceed these thresholds in the future were primarily clustered along state and U.S. highways east of Phoenix, with some localized potential impacts on connectivity pinch points across the state (Figures 3c and S3; Table S5).

## 4 Discussion

The model of puma habitat connectivity presented here identified large, contiguous areas of high connectivity across the state of Arizona, but also numerous movement and dispersal pinch points. These results are based on contemporary estimates of vegetation cover and the level of human influence on the region. Still, many areas were identified where an increase in the amounts of impervious surface and traffic volume are likely to create new or more expansive barriers, which may reduce connectivity. For example, although the natural areas between Phoenix, Prescott, and Payson exhibited some of the highest potential connectivity, these places may also be the most likely to experience intensified use by a growing human population. In fact, Maricopa County, which includes large portions of north and east Phoenix and its suburbs, as well as Yavapai County, which includes the city of Prescott, were two of the fastest growing counties in the U.S. in 2014 (U.S. Census Bureau 2015). Because Arizona also is one of the fastest growing states in the country (U.S. Census Bureau 2015), city planners and resource managers in Arizona have been working together to mitigate the adverse impacts of future development, including transportation corridors (Nordhaugen et al., 2006). Habitat conservation efforts should remain focused on intact natural areas, but should also seek to get ahead of expected future loss of connectivity due to growth of existing exurban areas at the wildland‐urban interface and resultant increases in traffic volume.

### 4.1 Future development impacts on connectivity pinch points

Pumas are sensitive to the presence of human structures and associated activities (Beier, 1995; Wilmers et al., 2013), particularly when artificially lit (Beier, 1995). While some authors have observed a functional response to housing density in which pumas occupying rural areas show higher tolerance for housing structures than pumas occupying more sparsely developed exurban areas (Burdett et al., 2010; Kertson, Spencer, & Grue, 2013), others have not detected this pattern (Wilmers et al., 2013) and tolerance is likely to only occur at very low housing densities. Despite the fact that pumas collared in this study were captured immediately adjacent to the urban centers of Tucson, Payson, and Prescott, they were rarely observed to pass through even the outer edges of these areas as defined by fairly abrupt increases in degree of human modification (also see Nicholson, 2009). Expansion of human settlements is generally expected to restrict puma movements and to reduce availability of high‐quality habitat required to support more sensitive behavioral states, particularly reproduction (Wilmers et al., 2013).

This study identified pinch points expected to be most vulnerable to development that could adversely impact puma movements based on projected impervious surface expansion (Figures 3a and S1; Table S3). In some cases, narrow pinch points are expected to be further constricted or to be completely severed as future development expands along major highways (e.g., Figure S1c,d). These sites may present opportunities to proactively protect critical corridors through private lands by securing conservation easements or wildlife‐friendly development practices (e.g., White & Penrod, 2012). In others, expansion at the fringes of existing large urban centers may impact adjacent high connectivity areas (e.g., Figure S1a,b). These sites may be subject to increasing human‐wildlife conflict and perhaps warrant proactive efforts to increase awareness and prevent such conflicts.

### 4.2 Current and future traffic impacts on connectivity pinch points

Arizona is crossed by >189,000 km of paved roads. Roads on which traffic are monitored carry average daily traffic volumes of up to 281,000 vehicles/day in urban centers, but average 13,000 vehicles/day across the state. Wildlife‐vehicle collisions (WVCs) are an important source of mortality for pumas. Beier and Barrett (1993) found that the leading cause of mortality (32%) for pumas in southern California was WVCs, and in five years of study (1988–1992), Dickson et al. (2005) observed only a single nonfatal freeway crossing. Taylor, Buergelt, Roelke‐Parker, Homer, and Rotstein (2002) observed the same pattern in Florida panthers: 35% of panther mortality from 1978 to 1999 involved collisions with vehicles, and 80% of these deaths were during the summer tourist season when traffic volumes increased.

In addition to WVC mortality, roads also have indirect effects on puma habitat connectivity. Pumas have been observed to avoid paved roads with two or more lanes (Dickson et al., 2005; Sweanor et al., 2000), and Schwab and Zandbergen (2011) observed that successful crossing frequency was inversely proportional to road class, with smaller roads being crossed more frequently. High traffic volume is understood to increase the barrier effect of a road (e.g., Alexander et al., 2005; van Langevelde & Jaarsma, 2004), but thresholds at which traffic becomes a complete barrier to movement are not well understood. In Banff National Park, Alberta, cougars frequently crossed a highway with 3,000 AADT, but did not cross the Trans‐Canada Highway with 14,000 AADT (Alexander & Waters, 2000). Alexander et al. (2005) suggest that in Banff, the threshold at which roads become a barrier to cougar movement is approximately 3,000–5,000 AADT.

Despite their avoidance of roads, pumas are known to use crossing structures (i.e., wildlife over‐ and underpasses), which have been demonstrated to promote demographic and genetic connectivity of large‐bodied, wide‐ranging mammals (e.g., Sawaya, Clevenger, & Kalinowski, 2013; Sawaya, Kalinowski, & Clevenger, 2014). In fact, 520 uses of Banff National Park crossing structures have been documented from 1996 to 2001 (Gloyne & Clevenger, 2001). Underpasses appear to be more effective for pumas than overpasses, and preferences for structure design may favor either very open structures (i.e., open span bridges: Beier, 1995; Gloyne & Clevenger, 2001) or long, narrow, more constricted passages (Clevenger & Waltho, 2005). Wing fencing that funnels pumas toward crossing structures is critical for their effectiveness (Schwab & Zandbergen, 2011). Beier (1995) emphasizes that pumas will not seek out crossings; rather, they will only use structures encountered along normal travel routes, and crossing structures near high quality habitat have been observed to have the highest rates of use (Clevenger & Waltho, 2005; Gloyne & Clevenger, 2001).

The model of puma connectivity presented here can augment other resources (e.g., Nordhaugen et al., 2006) in guiding selection of crossing structure sites that are most likely to be effective in enhancing puma habitat connectivity across high‐volume Arizona highways. This study highlights connectivity pinch points crossed by roads that carry high volumes of traffic (>5,000 AADT; Figures 3b and S2; Table S4), which are expected to create barriers to puma movement where opportunities for safe passage (e.g., wildlife underpasses) are not available, either due to deterrence from attempting to cross the road or WVC mortality. Road segments that are currently expected to have low impacts (<3,000 AADT) or marginal impacts (3,000–5,000 AADT), but that are projected to be used more heavily in the future (Figures 3c and S3; Table S5), may present opportunities for proactive wildlife crossing structure installation to prevent these roads from becoming absolute barriers to movement (Ament et al., 2014).

## 5 Conclusions

The empirically based, statewide estimate of connectivity presented here was qualitatively similar to the expert‐based model derived by Dickson, Roemer, et al. (2013) for Arizona and New Mexico. The present model, however, was trained with telemetry data collected in areas adjacent to Arizona's major urban centers. Following the recommendation of Dickson, Roemer, et al. (2013), this high‐resolution analysis of puma movement patterns, including sixteen young adults that may have exhibited dispersal‐related movements, provides the fine‐scale information needed for project‐level planning. In conjunction with spatial information regarding puma abundance and mortality, as well as consideration of the context of highlighted pinch points within a broader network of puma habitat (e.g., McRae, Hall, Beier, & Theobald, 2012, Dickson, Roemer, et al., 2013; Dickson, Sesnie, et al., 2013), results of this analysis can help guide prioritization of connectivity conservation actions. On public lands exhibiting high potential connectivity, these results can help to inform city, county, and state planning practices and development patterns that are sensitive to wildlife needs, including safe passages near urban centers. On private lands, these results can provide guidance, for example, to land trusts seeking to prioritize acquisition of easements on lands with high value for wildlife connectivity.

As changes to native land cover and expansion of human transportation corridors continue, pumas moving or dispersing across the state of Arizona are likely to encounter new or unfamiliar barriers. These barriers have the potential to cause individual mortality and affect regional meta‐population structure (McRae et al., 2005). Because the Arizona region has been subject to prolonged drought and is expected to experience dramatic changes in climate (Seager et al., 2007), the quality and potential connectedness of habitat may be further reduced. Resource managers, planners, and policy makers will need to be coordinated and targeted in their actions to preserve or restore connectivity in a rapidly changing environment. This may be particularly valuable and pertinent if the conservation of pumas and permeability of their habitat also protects the status of other species (e.g., Kunkel, Atwood, Ruth, Pletscher, & Hornocker, 2013).

## Data accessibility

All derived data products resulting from this analysis are to be publicly archived via Dryad. However, raw GPS collar data are held by a third party (AGFD) and permission to archive these data publicly has not been granted to the authors due to sensitivities surrounding the whereabouts of a large, predatory game species occupying home ranges adjacent to urban centers.

## Conflict of Interest

None declared.
