# Supplementary material for: Modeling connectivity to identify current and future anthropogenic barriers to movement of large carnivores: A case study in the American Southwest
Source: Ecol Evol. 2017 Apr 18;7(11):3762–72. doi: 10.1002/ece3.2939 (PMC5468141; doi:10.1002/ece3.2939)
Supplement: Supplementary file 1 [file ECE3-7-3762-s001.pdf]

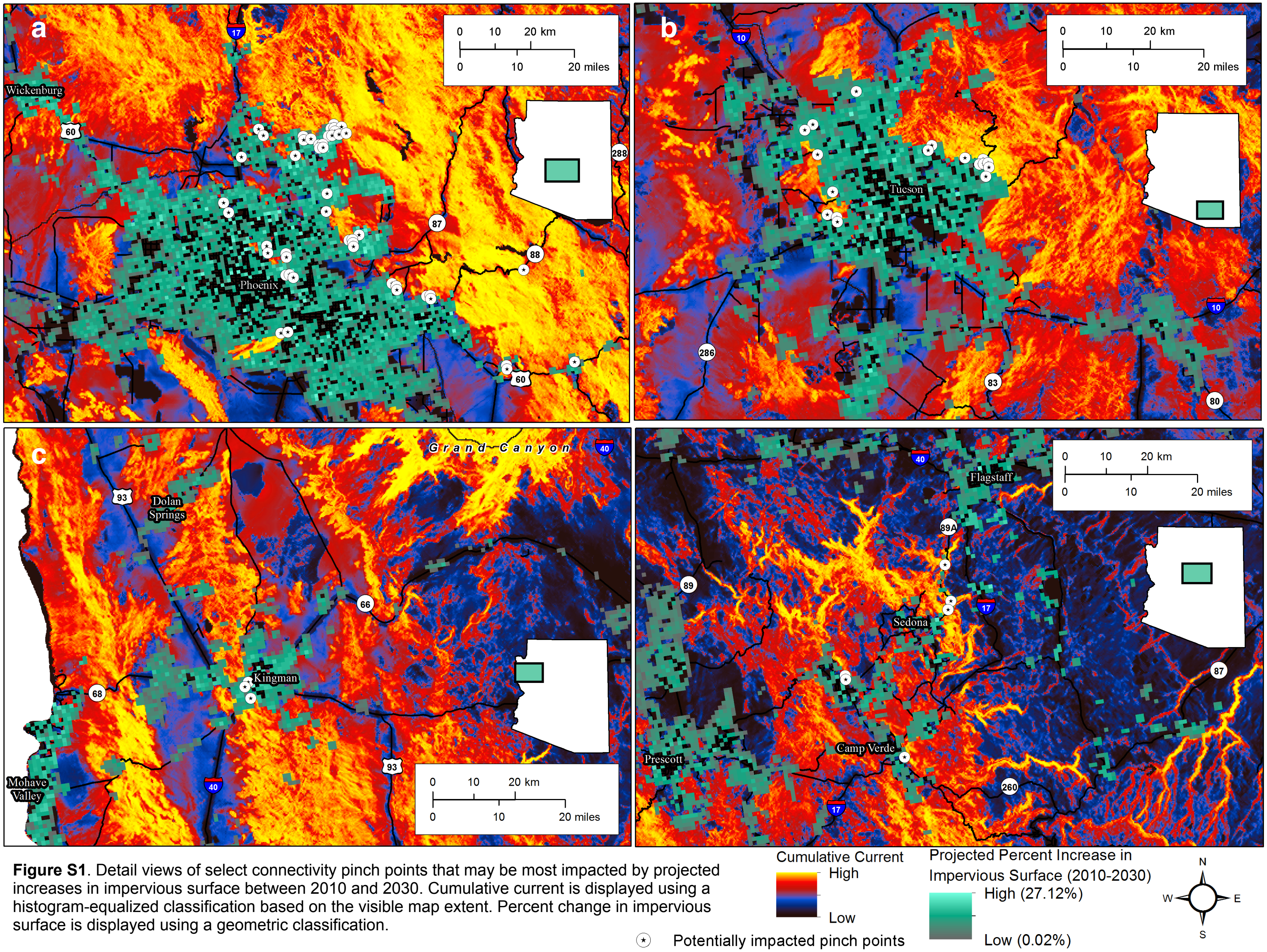

Figure S1. Detail views of select connectivity pinch points that may be most impacted by projected increases in impervious surface between 2010 and 2030. Cumulative current is displayed using a histogram-equalized classification based on the visible map extent. Percent change in impervious surface is displayed using a geometric classification.
