# Supplementary material for: Modeling connectivity to identify current and future anthropogenic barriers to movement of large carnivores: A case study in the American Southwest
Source: Ecol Evol. 2017 Apr 18;7(11):3762–72. doi: 10.1002/ece3.2939 (PMC5468141; doi:10.1002/ece3.2939)
Supplement: Supplementary file 2 [file ECE3-7-3762-s002.pdf]

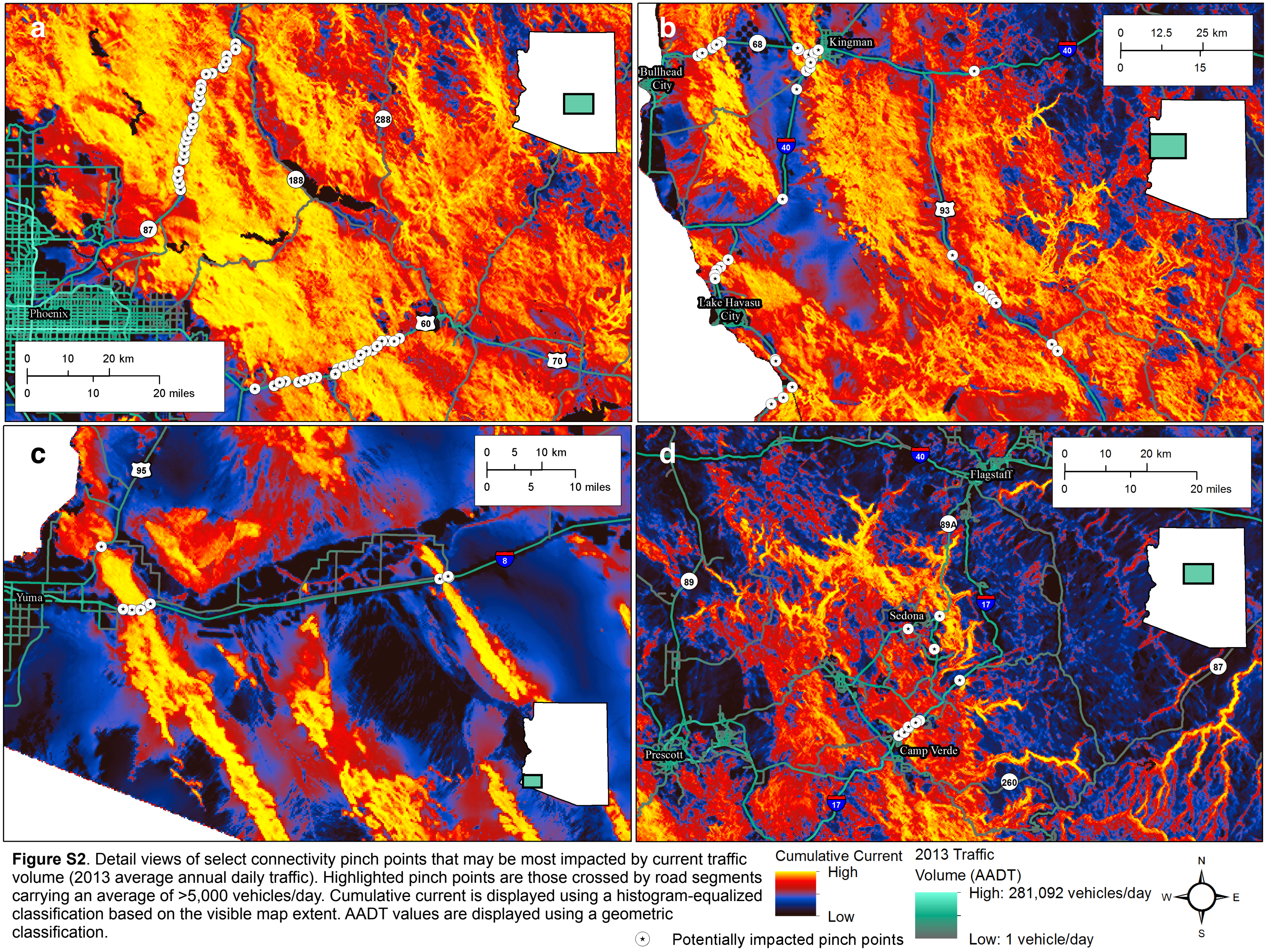

Figure S2. Detail views of select connectivity pinch points that may be most impacted by current traffic volume (2013 average annual daily traffic). Highlighted pinch points are those crossed by road segments carrying an average of >5,000 vehicles/day. Cumulative current is displayed using a histogram-equalized classification based on the visible map extent. AADT values are displayed using a geometric classification.
