# Supplementary material for: Modeling connectivity to identify current and future anthropogenic barriers to movement of large carnivores: A case study in the American Southwest
Source: Ecol Evol. 2017 Apr 18;7(11):3762–72. doi: 10.1002/ece3.2939 (PMC5468141; doi:10.1002/ece3.2939)
Supplement: Supplementary file 3 [file ECE3-7-3762-s003.pdf]

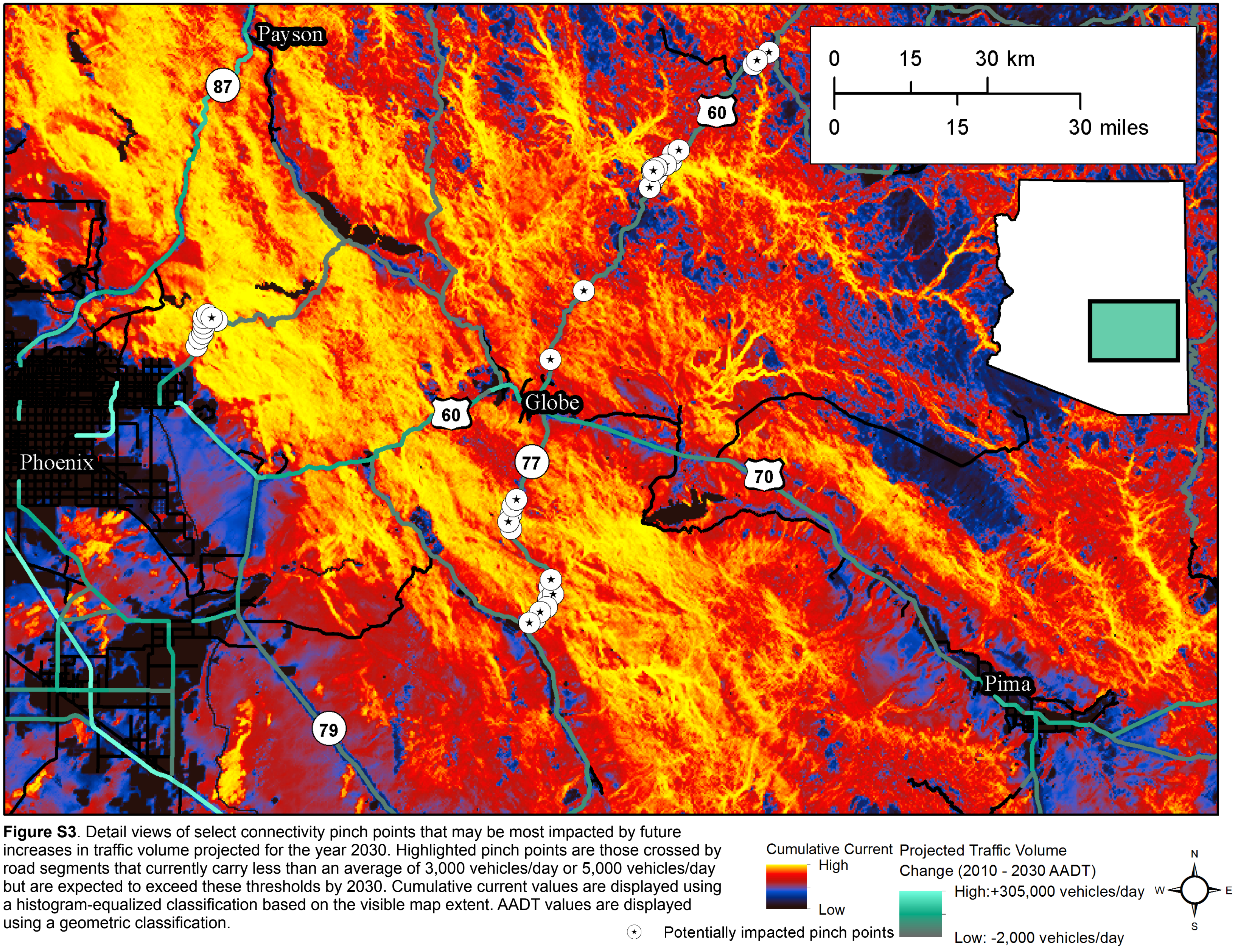

Figure S3. Detail views of select connectivity pinch points that may be most impacted by future increases in traffic volume projected for the year 2030. Highlighted pinch points are those crossed by road segments that currently carry less than an average of 3,000 vehicles/day or 5,000 vehicles/day but are expected to exceed these thresholds by 2030. Cumulative current values are displayed using a histogram-equalized classification based on the visible map extent. AADT values are displayed using a geometric classification.
