# Supplementary material for: Modeling connectivity to identify current and future anthropogenic barriers to movement of large carnivores: A case study in the American Southwest
Source: Ecol Evol. 2017 Apr 18;7(11):3762–72. doi: 10.1002/ece3.2939 (PMC5468141; doi:10.1002/ece3.2939)
Supplement: Supplementary file 4 [file ECE3-7-3762-s004.docx]

**Supporting Information**

McClure ML, Dickson BG, Nicholson KL. Modeling connectivity to identify current and future anthropogenic barriers to movement of large carnivores: a case study in the American Southwest.

**Table S1.** Descriptive statistics for the GPS collar dataset used to fit Brownian bridge movement models (BBMMs). Statistics were derived after removing data points with PDOP > 10, data from one collar that collected only 73 locations, and points that were < 200m from adjacent locations and thus were likely to represent activity around den or kill sites rather than bouts of active movement.

|  | *Mean* | *SD* | *Median* |
| --- | --- | --- | --- |
| Age at capture (years) | 4.7 | 2.2 | 3.5 |
| Tracking duration (days) | 344 | 156 | 330 |
| Location frequency (hours) | 8.5 | 56.9* | 7 |
| Total locations/individual | 725 | 337 | 736 |
|  |  |  |  |
| Sex |  | 7 F | 21 M |
| Capture location | 6 Payson | 9 Prescott | 13 Tucson |

* After excluding data points with lag > 24 hours when fitting BBMMs, SD = 3.6

**Table S2. Summary of habitat variables.** Description, source information, and supporting references for variables used to estimate habitat quality for puma movement.

| *Variable* | *Description* | *Source* | *Reference* |
| --- | --- | --- | --- |
| Ruggedness | Standard deviation of slope in 270 m focal area | Landfire DEM^1^ | Burdett et al. 2010; Hernandez-Santin, Harveson & Harveson 2012; Dickson et al. 2013 |
| Topographic Position Index (TPI) | Mean difference in focal elevation and mean elevation across multiple neighborhood extents | Theobald et al. 2015 | Dickson & Beier 2006; Dickson et al. 2013 |
| Distance to Water | Distance to nearest perennial water source or water source maintained for wildlife use | National Hydrography Dataset^2^, Arizona Wildlife Waters^3^, Southwest ReGAP^4^ | LaRue & Nielsen 2008; Kertson & Marzluff 2010; Dickson et al. 2013; Wilmers et al. 2013 |
| Percent Forest/ Woodland Cover | Percent of 270 m focal area with forest or woodland dominated cover types | Southwest ReGAP | Dickson & Beier 2002; LaRue & Nielsen 2008; Kertson et al. 2011; Dickson et al. 2013; Wilmers et al. 2013 |
| Percent Riparian Cover | Percent of 270 m focal area with riparian dominated cover types | Southwest ReGAP | Dickson & Beier 2002; LaRue & Nielsen 2008; Burdett et al. 2010; Dickson et al. 2013 |
| Percent Shrub/Scrub Cover | Percent of 270 m focal area with shrub or scrub dominated cover types | Southwest ReGAP | Dickson & Beier 2002; Dickson et al. 2013; Wilmers et al. 2013 |
| Human Modification | Multi-scale index of proportion of landscape within given neighborhood size that is human-modified | Theobald 2013 | Dickson & Beier 2002; Dickson, Jenness & Beier 2005; Dickson et al. 2013; LaRue & Nielsen 2008; Kertson et al. 2011; Wilmers et al. 2013 |
| ^1^USGS 2012; ^2^USGS 2014; ^3^Arizona Game and Fish Department; ^4^USGS National Gap Analysis Program 2004 | | | |

**References**

Burdett, C.L., Crooks, K.R., Theobald, D.M., Wilson, K.R., Boydston, E.E., Lyren, L.M., Fisher, R.N., Vickers, T.W., Morrison, S. a. & Boyce, W.M. (2010) Interfacing models of wildlife habitat and human development to predict the future distribution of puma habitat. *Ecosphere*, **1**, art4.

Dickson, B. & Beier, P. (2002) Home-range and habitat selection by adult cougars in southern California. *The Journal of Wildlife Management*, **66**, 1235–1245.

Dickson, B.G. & Beier, P. (2006) Quantifying the influence of topographic position on cougar (Puma concolor) movement in southern California, USA. *Journal of Zoology*, **271**, 270–277.

Dickson, B., Jenness, J. & Beier, P. (2005) Influence of vegetation, topography, and roads on cougar movement in Southern California. *Journal of Wildlife Management*, **69**, 264–276.

Dickson, B., Roemer, G., McRae, B. & Rundall, J. (2013) Models of regional habitat quality and connectivity for pumas (Puma concolor) in the southwestern United States. *PloS ONE*, **8**, e81898.

Hernandez-Santin, L., Harveson, P.M. & Harveson, L. a. (2012) Suitable habitats for cougars (Puma concolor) in Texas and Northern Mexico. *Southwestern Naturalist*, **57**, 314–318.

Kertson, B.N. & Marzluff, J.M. (2010) Improving studies of resource selection by understanding resource use. *Environmental Conservation*, **38**, 18–27.

Kertson, B., Spencer, R., Marzluff, J., Hepinstall-Cymerman, J. & Grue, C. (2011) Cougar space use and movements in the wildland-urban landscape of western Washington. *Ecological Applications*, **21**, 2866–2881.

LaRue, M. a. & Nielsen, C.K. (2008) Modelling potential dispersal corridors for cougars in midwestern North America using least-cost path methods. *Ecological Modelling*, **212**, 372–381.

Theobald, D.M. (2010) Estimating natural landscape changes from 1992 to 2030 in the conterminous US. *Landscape Ecology*, **25**, 999–1011.

Theobald, D.M., Harrison-Atlas, D., Monahan, W.B. & Albano, C.M. (2015) Ecologically-relevant maps of landforms and physiographic diversity for climate adaptation planning. *PLoS ONE*, **10**, e0143619.

USGS. (2012) LANDFIRE: LANDFIRE 1.3.0 Digital Elevation Model layer. URL http://www.landfire.gov/topographic.php

USGS. (2014) National Hydrography Dataset (NHD). URL http://datagateway.nrcs.usda.gov USGS National Gap Analysis Program. (2004) Provisional Digital Land Cover Map for the Southwestern

United States (Version 1.0). URL http://earth.gis.usu.edu/swgap/landcover.html Wilmers, C.C., Wang, Y., Nickel, B., Houghtaling, P., Shakeri, Y., Allen, M.L., Kermish-Wells, J.,

Yovovich, V. & Williams, T. (2013) Scale dependent behavioral responses to human development by a large predator, the puma. *PloS one*, **8**, e60590.
